# Non-small cell lung cancer treatment (r)evolution: ten years of advances and more to come

**DOI:** 10.3332/ecancer.2017.787

**Published:** 2017-11-30

**Authors:** Luca Toschi, Sabrina Rossi, Giovanna Finocchiaro, Armando Santoro

**Affiliations:** Humanitas Research Hospital, Medical Oncology, Via Manzoni 56, 20089 Rozzano, Italy

**Keywords:** non-small cell lung cancer (NSCLC), epidermal growth factor receptor (EGFR), anaplastic lymphoma kinase (ALK), program death 1 receptor (PD-1), checkpoint inhibitors

## Abstract

Diagnostic and treatment algorithms in non-small cell lung cancer (NSCLC) are evolving at a never-before-seen pace. Histological subtyping to maximise treatment efficacy and avoid toxicity has marked the beginning of the revolution, opening the way to molecular characterisation to guide genomically driven treatments with targeted agents, led by Epidermal Growth Factor Receptor (EGFR) and Anaplastic Lymphoma Kinase (ALK) inhibitors. More recently, agents against the Program Death 1 receptor (PD-1) and ligand 1 (PD-L1) have entered the clinical arena, offering new hope to NSCLC patients, although several uncertainties remain to be elucidated. Here, we review the most clinically relevant advances in the diagnosis and treatment of NSCLC in the past decade.

## Introduction

In 2002, the Eastern Oncology Cooperative Group (ECOG) published in the New England Journal of Medicine the results of a landmark randomised phase III trial comparing four chemotherapy platinum-based doublets in patients with untreated advanced non-small cell lung cancer (NSCLC) [1]. The rationale for conducting such a trial was to gain an insight into the efficacy of new, at that time, platinum partners, such as taxanes, gemcitabine and vinorelbine. The trial showed no overall survival (OS) difference between the four regimens, providing a clear take-home message for clinicians: in advanced NSCLC patients, any platinum-based doublet with third-generation agents can be used, with no need to refine patient selection based on clinical characteristics other than good performance status (ECOG PS 0–1).

In the past decade, treatment algorithms for NSCLC have dramatically evolved and are increasingly branching out as a result of better clinical and biological patient selection, availability of new agents (as indicated in Tables 1 and 2) and some amount of serendipity.

## Histology matters: lessons about toxicity and efficacy

In the abovementioned ECOG trial, data on histology were not reported. In fact, in 2002, identification of NSCLC histological subtypes was considered irrelevant, with pathologists being required to discriminate only between NSCLC and small-cell lung cancer (SCLC), the latter following a peculiar treatment algorithm. The first hints suggesting that histological subclassification of NSCLC could play a key role in treatment decision emerged from a randomised phase II trial investigating the efficacy and safety of bevacizumab, a humanised monoclonal antibody directed against the vascular endothelial growth factor (VEGF), in combination with carboplatin and paclitaxel in patients with advanced NSCLC [2]. While the experimental arm showed promising signs of increased efficacy over chemotherapy alone, with improved response rate (RR), time-to-progression and OS, 6/66 (9%) bevacizumab-treated patients experienced severe bleeding events, mostly hemoptysis, with four being fatal. Importantly, the increased bleeding risk was associated with squamous cell histology and this observation led to the subsequent development of bevacizumab exclusively in nonsquamous NSCLC. In another pivotal randomised phase III ECOG trial, the combination of carboplatin, paclitaxel and bevacizumab was compared with chemotherapy alone in a histologically selected population, with only nonsquamous NSCLC patients being included, and led to an impressive two-month improvement in OS, for the first time passing the 12-month mark [3]. Based on the results of this trial, the use of bevacizumab took hold in the United States, where carboplatin and paclitaxel was a commonly used chemotherapy regimen. In contrast, the AVAiL study failed to show an increased OS for bevacizumab in addition to cisplatin and gemcitabine [4], a popular chemotherapy regimen in Europe, somehow limiting bevacizumab use out of the United States. More recently, attempts have been made to redeem antiangiogenic agents in pretreated squamous cell NSCLC with contrasting results. On one hand, ramucirumab, a human IgG1 antiVEGFR2 monoclonal antibody, improved OS in combination with docetaxel in unselected NSCLC, including squamous cell histology, without increasing the incidence of severe bleeding events [5]. In contrast, the use of nintedanib in the same setting, while active in lung adenocarcinoma, failed to improve OS over single-agent docetaxel in patients with squamous cell cancer, albeit with no increased major bleeding toxicity [6].

Other clear signals about the crucial relevance of an accurate histological NSCLC characterisation came with the development of pemetrexed, an antifolate multitarget agent, which is currently used in the first- and second-line treatment of nonsquamous NSCLC. In the H3E-MC-JMDB, noninferiority phase III trial 1725 advanced NSCLC patients were randomised to cisplatin-gemcitabine or cisplatin-pemetrexed [7]. The trial met its primary endpoint of noninferior OS for the pemetrexed versus gemcitabine-based arm; however, subgroup analyses revealed differential activity of the two regimens based on histology. In fact, OS was significantly longer for cisplatin-pemetrexed versus cisplatin-gemcitabine in patients with adenocarcinoma (12.6 vs 10.9 months; HR 0.84, p = 0.03) and large-cell carcinoma histology (10.4 vs 6.7 months; HR 0.67, p = 0.03). Conversely, in patients with squamous cell histology, OS was significantly improved with cisplatin-gemcitabine versus cisplatin-pemetrexed (10.8 vs 9.4 months; HR 1.24, p = 0.05). Similarly, a *post hoc* analysis conducted within a phase III trial of pemetrexed versus docetaxel as second-line treatment in unselected NSCLC showed the differential activity of the two agents according to histology [8], leading to pemetrexed approval and future development in nonsquamous tumours only.

More recent examples of new agents developed in histologically selected NSCLC populations with different results include, among others, the antiepidermal growth factor receptor (EGFR) necitumumab. When added to cisplatin and pemetrexed in the phase III INSPIRE trial, which enrolled patients with nonsquamous histology only, necitumumab failed to prolong survival but increased toxicity, including serious adverse events [10]. In contrast, the SQUIRE trial, which investigated cisplatin and gemcitabine with or without necitumumab in squamous cell histology, met its primary endpoint of improved OS for the experimental arm (11.5 vs 9.9 months; HR 0.84, p = 0.01) [11]. A *post hoc* analysis showed that the benefit was limited to patients with some degree of EGFR protein expression assessed by immunohistochemistry (IHC) [12], leading to necitumumab approval by the US Food and Drug Administration (FDA) and European Medicines Agency (EMA) in EGFR-expressing squamous cell NSCLC.

Overall, the above-discussed data highlight the key role of histology in clinical practice and in the development of new agents, both for safety concerns and efficacy. As a result, the past decade has seen a progressive refinement of the diagnostic path in NSCLC, with massive efforts to obtain adequate samples for an accurate histological characterisation of the disease.

## Oncogene-addicted NSCLC: the quest for molecular targets

Within the past several years, improvement of molecular biology techniques contributed to the identification of specific mutated oncogenes in NSCLC for which therapeutic treatments have either become commercially available or are under investigation, leading to a paradigm shift in the treatment of NSCLC. Therefore, appropriate tumour sampling is essential not only for histological subtyping but also for accurate tissue genotyping, as highlighted by the ‘tissue is the issue’ slogan coined few years ago.

### EGFR gene mutations

Even before the crucial role of histology was established, the first clinically relevant molecular target in NSCLC was being identified, the *EGFR* gene mutations. If we look now at the major use of EGFR tyrosine kinase inhibitors (TKIs) in clinical practice, the whole EGFR story started with a fair amount of serendipity coupled with brilliant scientific intuitions from academic researchers. In fact, gefitinib and erlotinib – the first two oral EGFR TKIs to have reached the clinical arena – were initially investigated either alone or in combination with chemotherapy in unselected NSCLC populations based on the observation that the vast majority of lung tumour cells express EGFR on their surface [13]. Combination phase III trials of TKIs with platinum-based chemotherapy turned out negative for both drugs [14–17], while single-agent gefitinib showed some efficacy in terms of RR in the phase II IDEAL 1 and 2 trials [18, 19]. In 2003, the FDA approved gefitinib after chemotherapy failure in unselected NSCLC, but required a phase III trial to investigate gefitinib effect on survival – the ISEL study, which failed to show an OS improvement for gefitinib versus placebo [20] and led the FDA to withdraw its previous approval. However, in the years before FDA gefitinib registration, several patients could receive the drug within and expanded access programme, with clinicians observing major responses in a minority of patients, mostly women, nonsmokers and with adenocarcinoma. In the attempt of identifying the molecular bases underlying gefitinib activity in these subgroups, researchers sequenced *EGFR* exons in major responders, identifying what today are known as sensitising, not only to gefitinib but also to other TKIs, mutations in the tyrosine kinase domain, with exon 19 deletions and the L858R point mutation in exon 21 being the most frequent [21, 22]. Preclinical data were generated to confirm such findings, and a number of randomised trials comparing TKIs with platinum-based chemotherapy were performed in *EGFR*-mutated patients, invariably showing a dramatically superior progression-free survival (PFS) in favour of TKIs (9–13 vs 5–7 months) with RR of about 60–70% [23–28]. In short, gefitinib (which has been reapproved by the FDA) and erlotinib (both first-generation TKIs) and, more recently, afatinib (second-generation TKI) are nowadays first-line options in several countries for patients with *EGFR*-mutant NSCLC, and as a result, clinical guidelines are largely concordant in recommending *EGFR* mutation analysis in all patients with advanced nonsquamous histology regardless of clinical characteristics. Clinicians should review EGFR testing results before commencing any systemic treatment; therefore, it is crucial to have a quick turnaround time for the analysis. But how many patients are expected to be eligible for first-line TKIs? Interestingly while sensitising, *EGFR* mutations are identified in about 10%–15% of Caucasian patients, they occur in up to 60% of Asian populations with NSCLC [29], and the reason for such a difference remains largely unknown.

Despite the initial benefit from EGFR TKIs, patients will ultimately develop acquired resistance, which, in up to 60% of cases, is mediated by the development of a secondary mutation in exon 20, the T790M, as shown by rebiopsies performed at the time of disease progression [30]. The T790M mutation causes treatment failure by increasing adenosine triphosphate (ATP) affinity within the ATP-binding pocket in the EGFR tyrosine kinase domain rather than sterically blocking drug binding [31]. Understanding of the T790M biology prompted the development of agents that can selectively bind to the T790M mutant allele with a minimal activity against wild-type *EGFR*, which is responsible for toxicity caused by first- and second-generation TKIs [32]. Recently, osimertinib, a third-generation EGFR TKI, has been approved by FDA and EMA for patients with acquired resistance to first- and second-generation TKIs mediated by the T790M mutation. In the phase II single-arm AURA-2 study, osimertinib showed antitumour efficacy in T790M-mediated acquired resistance in patients pretreated with EGFR TKIs with an RR of 62% and a PFS of 12.3 months [33]. More recently, the drug was compared with platinum-pemetrexed chemotherapy in T790M-positive patients after first-line TKI failure in the AURA-3 trial [34]. The drug showed improved PFS (10.1 vs 4.4 months, p < 0.001) and RR (71% vs 31%, p < 0.001), and the benefit was confirmed in patients with brain metastases. The drug has also showed promising activity against central nervous system (CNS) metastases, including leptomeningeal disease [35]. Overall, these data clearly indicate that the T790M mutation should be carefully sought in *EGFR*-mutant patients progressing under first-line TKIs. Today, we know that the test can be reliably performed with a simple blood test by analysing cell-free DNA and, more recently, urine samples have been deemed suitable as well, with sensitivity >70% [36, 37]. Based on these data, rebiopsy could be limited only to patients with a T790M-negative blood/urine test.

Soon after osimertinib become available in several countries for T790M-mediated resistance on first- or second-generation TKI, data from the eagerly awaited phase III FLAURA trial showed that osimertinib is clearly superior, in terms of PFS, over gefitinib or erlotinib in the first-line setting in patients with common *EGFR* mutations (18.9 vs 10.2 months, HR 0.46; p < 0.0001), with a trend for better OS [38]. These findings led to Breakthrough Therapy Designation by FDA and will likely establish first-line osimertinib as a new standard of care for *EGFR*-mutant NSCLC, limiting the penetration in clinical practice of more recently developed strategies for TKI-naïve patients such as dacomitinib, a second-generation TKI [39], or the combination of erlotinib and bevacizumab [40].

The key issue will now be to elucidate mechanisms of acquired resistance to first-line osimertinib and understand how to best to treat patients at disease progression.

### Anaplastic Lymphoma Kinase (ALK) rearrangements

ALK is the second target after EGFR to have reached clinical relevance in NSCLC, after its discovery in 2007 [41], with an increasing number of ALK inhibitors becoming available in clinical practice. ALK is a member of the insulin receptor superfamily of tyrosine kinases. Chromosomal rearrangements of *ALK* are present in 3%–7% of NSCLCs and are associated with adenocarcinoma histology, lower age at diagnosis and never/light smoker status [42]. The resulting *ALK* fusions, such as *EML4-ALK*, showed responsiveness of ALK-positive tumours to ALK TKIs [43].

Crizotinib is an oral inhibitor of ALK currently approved in several countries for the treatment of ALK-positive and, more recently, ROS1-positive NSCLCs. It showed a statistically and clinically significant improvement of RR and PFS compared with chemotherapy either in pretreated ALK-positive patients and, more importantly, in the first-line setting [44, 45]. Particularly, in treatment-naïve patients, responses were observed in 74% of those receiving crizotinib, with an impressive PFS of 10.9 months. Based on these findings, detection of *ALK* rearrangements with either fluorescence *in situ* hybridisation (FISH) or IHC has become part of the diagnostic workout of patients with nonsquamous histology [46]. Similarly to *EGFR*-mutant tumours progressing under TKIs, patients harbouring *ALK* rearrangements will ultimately acquire resistance to crizotinib due to a variety of mechanisms, with secondary *ALK* mutations representing the most investigated [47], and with CNS as one common site of metastatisation [48]. Thus, second- and third-generation ALK inhibitors have been developed to overcome acquired resistance to crizotinib, each with its own spectrum of activity against *ALK* mutations [49]. Ceritinib, alectinib and, more recently, brigatinib have all been approved by FDA in crizotinib-resistant patients, with no requirement for the identification of the mechanism responsible for crizotinib failure.

In the phase II ASCEND-2 trial, ceritinib was tested in crizotinib-refractory patients obtaining an encouraging RR of 38.6% and a PFS of 5.7 months and with a substantial intracranial activity [50]. The drug is active also in crizotinib-naïve patients, as shown by a recent randomised phase III clinical trial, ASCEND 4, where the comparator arm was platinum/pemetrexed chemotherapy and where PFS of patients receiving ceritinib reached a never-before-seen value of 16.6 months [51]. These data suggest that ceritinib could be potentially more active than crizotinib in patients with ALK-positive tumours naïve to TKIs, despite the absence of head-to-head randomised studies.

Similarly to ceritinib, alectinib showed significant activity in patients refractory to crizotinib in phase II trials (NP28673 and NP28761 studies) [52–54], but a different development strategy has been pursued in the TKI-naive setting. In fact, alectinib has been compared with crizotinib in two phase III randomised trials, J-ALEX, conducted in Japanese patients [55], and ALEX, a global study [56], with PFS as the primary endpoint. Both trials showed superior PFS for alectinib versus crizotinib with HR 0.34, p < 0.0001 in J-ALEX and HR 0.47, p < 0.001 in ALEX. Based on these results, alectinib has been recently approved by the FDA as a first-line treatment in ALK-positive NSCLC, leading one to wonder whether there will still be room for crizotinib in the treatment algorithm of patients with *ALK* rearrangements.

More recently, brigatinib was granted FDA approval based on results from the phase I/II ALTA trial, where RR was 72% (51/71) in the cohort of crizotinib-resistant patients, with a favourable toxicity profile and with the evidence of promising intracranial activity [57].

Several other ALK TKIs are under development, including lorlatinib, ensartinib and entrectinib, to mention a few. Only time and, most of all, clinical trials will tell us which agent(s) will make it to the clinical arena and how to incorporate each molecule in the treatment strategy of ALK-positive NSCLC. Indeed, there is an evidence that the sequential use of different ALK TKIs could be beneficial to some patients [58], but a better knowledge of resistance mechanisms will likely be crucial to establish which agent to use and when.

### Beyond EGFR and ALK: novel actionable targets

Besides *EGFR* gene mutations and *ALK* gene rearrangements, which should be tested upfront in all patients with advanced nonsquamous NSCLC, other actionable targets have been recently identified and new genomic-driven agents are expanding the therapeutic arsenal against NSCLC. We will briefly review the targets with higher therapeutic interest and/or with potential clinical implementation in the next few years.

ROS1 is a receptor tyrosine kinase that has high homology to ALK [59]. *ROS1* gene fusions are found in approximately 2% of NSCLCs, seem to be mutually exclusive with other oncogenic driver genes and have been associated with nonsmoking history [60]. The high homology between ROS1 and ALK renders *ROS1*-rearranged tumours sensitive to several ALK inhibitors. Crizotinib demonstrated a potent inhibitory activity against ROS1 in a single-arm study with a median PFS of 19.2 months and an RR of 72% [61], leading to FDA and EMA approval for patients with *ROS1*-rearranged NSCLC.

BRAF is a well-established target in melanoma, where the commonly observed *V600E* point mutation leads to constitutive BRAF signalling and sensitivity to BRAF inhibitors [62]. More recently promising results have been observed in *BRAF*-mutant NSCLC. *BRAF* mutations occur in 1.6%–3% of NSCLCs and *V600E* represents about half of cases [63]. The *V600E* mutation has been associated with a more aggressive behavior, with poorer prognosis and is more common in females and less associated with smoking history [63, 64]. Dabrafenib and vemurafenib demonstrated promising single-agent efficacy in advanced NSCLC with *V600E BRAF* mutation, with an RR of 40%–42% in phase II trials [65, 66]. However, as in melanoma, drug combinations appear to yield increased efficacy in *V600E BRAF*-mutant NSCLC, with dabrafenib plus trametinib, an MEK inhibitor, showing an impressive RR of 63% and a PFS of 9.7 months [67]. This combination regimen was recently granted FDA Breakthrough Therapy Designation and received a recommendation for approval by the EMA Committee for Medicinal Products for Human Use (CHMP).

*RET* gene rearrangements have been firstly identified in papillary thyroid cancer and, more recently in about 1%–2% of unselected NSCLC [68]. In the phase II LURET trial, 17 *RET*-rearranged NSCLC were treated with vandetanib, a multitargeted TKI, with 53% RR and a relatively short, if compared with other genomic-driven treatment in NSCLC, median PFS of 4.7 [69]. No better results were obtained with cabozantinib in the same setting, with a 28% RR in 25 patients [70]. The hard time clinicians are experiencing in treating RET-positive NSCLC was recently confirmed by a retrospective analysis of 53 patients with *RET*-rearranged NSCLC treated with different RET-inhibitors, with RR up to 37% and median PFS of only 2.3 months [71]. These findings highlight the need for a better understanding of the biology of *RET*-rearranged lung cancers to improve outcomes for these patients.

The MET/hepatocyte growth factor (HGF) pathway has been identified as a potential therapeutic target in multiple solid tumours, including NSCLC [72]. Aberrant MET activation can be caused by several altered biological processes, but mainly two are of clinical interest in NSCLC: *MET* gene amplification, which is observed in 2%–4% of NSCLC, and *MET* exon 14 skip mutations, which have been found in 3%–4% of lung adenocarcinomas [73, 74]. Several agents with antiMET activity, including crizotinib, cabozantinib and capmatinib, have shown some degree of efficacy in patients with *MET*-amplified or mutant (exon 14 skipping) NSCLC, and some new agents are under investigation in this setting [75].

## Checkpoint inhibitors: the unexpected comeback of immunotherapy

In the era of precision medicine, where genomically driven-treatments are paving the way for the present and future of NSCLC, immunotherapy has made an unexpected, for many, comeback after a number of clinical failures. In fact, some degree of disbelief in the role of immunotherapy in NSCLC has arisen after large phase III trials failed to demonstrate a survival advantage for lung cancer vaccines over placebo in the adjuvant setting or as maintenance strategy in patients undergoing chemoradiation for locally advanced disease [76, 77]. Nevertheless, research efforts have been rewarded and between 2015 and 2016, four randomised phase III trials showed a clinically meaningful survival advantage for antiPD1 and PD-L1 agents (nivolumab, pembrolizumab and atezolizumab) over docetaxel in the second- and third-line setting [78–81], leading to the registration of these checkpoint inhibitors by the FDA and other regulatory agencies. This was just the beginning, or rather the close postbeginning – given the earlier success in melanoma – of the immunotherapy revolution, with several pharmaceutical companies now developing their own checkpoint inhibitor(s), not only in NSCLC.

Why are checkpoint inhibitors becoming so successful in cancer, with now several FDA-approved indications in solid tumours over the past few years? In the early phases of cancer development, a competent immune system destroys transformed cells, but through a variety of mechanisms, including expression of immunosuppressive factors like PD-L1, cancer cells can escape immune system surveillance [82]. In fact, PD-L1, expressed on the surface of tumour and infiltrating immune cells with immunosuppressive activity, interacts with PD-1, which is present on activated T-cells, causing immune escape. PD-1 and PD-L1 checkpoint inhibitors are monoclonal antibodies that disrupt PD-1/PD-L1-mediated signalling restoring antitumour immunity. Looking at the often impressive results from clinical trials with checkpoint inhibitors, this mechanism of immune escape occurs across several human malignancies, although patient selection remains a largely unresolved issue.

The first phase III positive trial in advanced NSCLC was CheckMate-017, where nivolumab, a fully human IgG4 antiPD1 monoclonal antibody, was compared with docetaxel as the second-line treatment in patients with squamous cell histology [78]. Median OS was significantly improved for nivolumab (9.2 vs 6.0 months; HR 0.59, p < 0.001), as well as RR (20% vs 9%, p = 0.008) and median PFS (3.5 vs 2.8 months; HR 0.62, p < 0.001). These results were partly replicated in the CheckMate-057 trial, performed in patients with nonsquamous histology, where nivolumab yielded superior median OS over docetaxel (12.2 vs 9.4 months; HR 0.73, p = 0.0015) [79]. The two CheckMate studies were closely followed by two other positive phase III trials, again versus docetaxel, with pembrolizumab [80], a humanised antiPD1 IgG4 monoclonal antibody, which has been developed as single agent exclusively in PD-L1-expressing tumours, and atezolizumab [81], a humanised IgG1antibody against PD-L1. Overall, these agents have an extremely favourable toxicity profile, with grade 3–4 adverse events occurring in no more than 15% of patients (vs 40%–50% with docetaxel) and with immune-related toxicity being generally manageable with steroids.

While in several countries PD-1/PD-L1 checkpoint inhibitors are/were yet to become available for pretreated patients, a groundbreaking trial opened the path for immunotherapy in first line. In fact, in the phase III KEYNOTE-024 trial, untreated subjects with advanced NSCLC whose tumours expressed high levels of PD-L1 (tumour proportion score, TPS, ≥ 50%), which represent about 1/3 of the screened population, were randomised to pembrolizumab or platinum-based chemotherapy [83]. The study showed a clear superiority of pembrolizumab in terms of PFS (median 10.3 vs 6.0 months; HR 0.50, p < 0.001), which was the primary endpoint, and OS (HR 0.60; p = 0.005), leading most guidelines to recommend PD-L1 testing in the diagnostic workout of patients with advanced NSCLC.

While the first-line algorithm looks relatively linear, the use of PD-1/PD-L1 inhibitors in the second (or third) line is generating much discussion. In fact, despite being welcomed with great enthusiasm by several clinicians, patients and media, checkpoint inhibitors cannot be considered the best option for every subject with advanced pretreated NSCLC. A major matter of debate is whether testing for PD-L1 expression, with the exception of pembrolizumab prescription, which requires a PD-L1-positive test (TPS ≥ 1%), could be a useful tool for patient selection, at least for nonsquamous histology (in the CheckMate-017 in squamous histology, PD-L1 was not predictive for nivolumab benefit). In fact, while several data suggest that RR, PFS and OS increase with PD-L1 levels [79–81, 84], there are patients with PD-L1 negative tumours who can still achieve a durable benefit from PD-1/PD-L1 inhibitors [79], indicating that a PD-L1-negative test should not necessarily exclude a patient from treatment. A still open question is how to reliably test for PD-L1 expression and if results from clinical trials are reproducible, given the different assays and cut-offs explored in each drug development, although harmonisation studies have been carried on and results are out there to let clinicians and pathologists make their choice [85, 86]. Emerging data suggest that novel potential markers could add to the complex picture of patient selection, including tumour mutation burden [87], but none of these looks broadly applicable in clinical practice in the short term. Indeed, some clinical and biological characteristics could aid the clinician in treatment decision making. For example, never-smoking history or presence of *EGFR* mutations or *ALK* rearrangements are among the subgroups less likely to benefit from anti-PD-1/PD-L1 agents [79–81], likely as a result of lower mutational burden [88], although the small number of patients with these characteristics included in clinical trials and the inevitably large confidence intervals should call for some caution and further investigation. At the same time, we should acknowledge that a substantial fraction of patients progressing under first-line platinum-based chemotherapy is unfit for triweekly docetaxel. This observation, together with the absence of subgroups where immunotherapy is overtly detrimental, may lead clinicians to opt for checkpoint inhibitors for all patients unfit for docetaxel, with potential overuse – also considering that the optimal treatment duration has not been defined yet – of these agents and with the risk of a significant economic impact on national health systems.

## Conclusion

Treatment of NSCLC is witnessing years of exciting progress with tangible improvements of patient outcome. The success of genomically driven treatments for small subgroups of patients highlights the need to test for selected molecular alterations in the clinical setting and calls for the identification of novel actionable targets. In parallel, clinical research should move forward from the traditional phase I–III drug development model, at least in the setting of oncogene-addicted tumours, promoting biomarker-driven studies, such as basket and umbrella trials, to grant timely access to new active agents to patients.

Additionally, as more and more agents become available, it will be increasingly crucial to understand how to best sequence treatments. To achieve this goal, clinicians and researchers should strive to systematically characterise mechanisms of resistance, with liquid biopsy potentially representing an ideal ally, given the noninvasiveness of sample collection and the ability of next-generation sequencing approaches to detect actionable targets on circulating tumour DNA [89].

In contrast, in the era of precision medicine, we are experiencing the paradox of immunotherapy, with the lack of reliable predictors of benefit and, more importantly, resistance, therefore drifting away from treatment personalisation. Indeed, these agents can produce durable benefit in NSCLC patients with diverse clinical characteristics and with long-term survival rates exceeding those of historical controls [90], but patient selection requires further refinement. The favourable safety profile of checkpoint inhibitors led to several ongoing clinical trials currently exploring combination strategies (i.e. checkpoint inhibitors with chemotherapy or targeted agents or multiple immune-oncology agents together) in order to tackle either intrinsic or acquired resistance, sometimes with limited preclinical evidence of synergistic effects. In this rush to develop novel treatment strategies, the substantial risk of added toxicity and the almost certain financial burden should be taken into account.

Finally, there is an urgent need to translate the improvements observed in advanced disease into the curative setting, where little progress, if any, has occurred in the past decade. The recent striking PFS benefit showed by the anti-PD-L1 agent durvalumab when compared with placebo as maintenance treatment after chemoradiation for stage III disease [91] is noteworthy and hopefully will increase cure rates in a setting where targeted agents have failed so far. Similarly, much hope is placed in the ongoing clinical trials with checkpoint inhibitors in the adjuvant setting, where the success rate of conventional treatments remains largely suboptimal.

## Figures and Tables

**Table 1.** FDA-approved targeted agents and angiogenesis inhibitors in NSCLC.

Drug	Class	Year	Indication(s)
Bevacizumab	AntiVEGF monoclonal antibody	2006	In combination with carboplatin and paclitaxel, for metastatic nonsquamous NSCLC
Erlotinib	First generation EGFR TKI	2013	*EGFR*-mutant (deletion exon 19 or L858R) advanced NSCLC
Afatinib	Second generation EGFR TKI	2013	*EGFR*-mutant (deletion exon 19 or L858R) advanced NSCLC
Ramucirumab	AntiVEGFR2 monoclonal antibody	2014	In combination with docetaxel for the treatment of patients with metastatic NSCLC progressing on or after platinum-based chemotherapy
Ceritinib	Second generation ALK/ROS1 TKI	2014	ALK-rearranged advanced NSCLC progressing on or intolerant to crizotinib
Necitumumab	AntiEGFR monoclonal antibody	2015	In combination with gemcitabine and cisplatin for first-line treatment of metastatic squamous NSCLC
Gefitinib	First generation EGF TKI	2015	*EGFR*-mutant (deletion exon 19 or L858R) advanced NSCLC
Osimertinib	Third generation EGFR TKI	2015	EGFR T790M+ advanced NSCLC progressing on or after EGFR TKI therapy
Crizotinib	First generation ALK/ROS1 TKI	2011 2016	ALK-rearranged advanced NSCLC ROS1-rearranged advanced NSCLC
Alectinib	Second generation ALK TKI	2015 2017	ALK-rearranged advanced NSCLC progressing on or intolerant to crizotinib First line ALK-rearranged advanced NSCLC
Brigatinib	Second generation ALK TKI	2017	ALK-rearranged NSCLC progressing on or intolerant to crizotinib

VEGF: Vascular Endothelial Growth Factor

VEGFR2: Vascular Endothelial Growth Factor Receptor-2

EGFR: Epidermal Growth Factor Receptor

ALK: Anaplastic Lymphoma Kinase

ROS1: ROS Proto-oncogene-1

TKI: tyrosine kinase inhibitor

NSCLC: non-small cell lung cancer

**Table 2.** FDA-approved PD-1/PD-L1 inhibitors in NSCLC.

Drug	Class	Year	Indication(s)
Nivolumab	AntiPD-1 monoclonal antibody	2015	Metastatic NSCLC progressing on or after platinum-containing chemotherapy
Pembrolizumab	AntiPD-1 monoclonal antibody	2015 2016 2017	Metastatic PD-L1-expressing (TPS ≥ 1%) NSCLC progressing on or after platinum-containing chemotherapy Metastatic PD-L1-expressing (TPS ≥ 50%) NSCLC, with no EGFR or ALK genomic tumour aberrations, and no prior systemic chemotherapy treatment for metastatic NSCLC Metastatic nonsquamous NSCLC in combination with pemetrexed and carboplatin, as first-line treatment
Atezolizumab	AntiPD-L1 monoclonal antibody	2016	Metastatic NSCLC progressing on or after platinum-containing chemotherapy

NSCLC: non-small cell lung cancer

TPS: tumour proportion score

EGFR: Epidermal Growth Factor Receptor

ALK: Anaplastic Lymphoma Kinase
